# Diet type and the oral microbiome

**DOI:** 10.3389/fnut.2025.1691952

**Published:** 2026-01-16

**Authors:** Daniel Betancur, Evelyn L. Jara, Celia A. Lima, Monserrat Victoriano

**Affiliations:** 1 Department of Surgical Stomatology, Faculty of Dentistry, Universidad de Concepción, Concepción, Chile; 2 Department of Pharmacology, Faculty of Biological Sciences, Universidad de Concepción, Concepción, Chile; 3 Department of Prevention and Public Dental Health, Faculty of Dentistry, Universidad de Concepción, Concepción, Chile; 4 Department of Nutrition and Dietetics, Faculty of Pharmacy, Universidad de Concepción, Concepción, Chile

**Keywords:** caloric restriction, diet, lifestyle, oral immunology, oral microbiome, vegan diet

## Abstract

**Background:**

The oral microbiome changes across the lifespan and is modulated by behavioral and metabolic exposures. Tobacco consumption, suboptimal hygiene, and frequent sugar intake disrupt microbial homeostasis, thereby increasing vulnerability to chronic oral diseases. While diet influences systemic metabolic and inflammatory health, evidence describing persistent, direct ecological effects on oral microbial communities remains limited.

**Objective:**

The objective of this study is to synthesize mechanistic insights on how dietary patterns shape the oral microbiome and influence systemic inflammatory or metabolic risk.

**Methodology:**

A narrative, non-systematic review was conducted through expansive literature exploration. Peer-reviewed original and clinical studies reporting defined dietary exposures caloric restriction, plant-based diets, inorganic nitrate and fiber intake, and high-fat or high-sugar processed diets, were qualitatively evaluated for mechanistic relevance.

**Key findings:**

Plant-enriched, high-fiber diets, nitrate intake, and caloric restriction were associated with reduced oxidative stress, lower pro-inflammatory cytokines, and greater diversity of commensal taxa, suggesting improved ecological stability. In contrast, processed diets promote metabolic conditions that indirectly remodel the oral habitat, favoring dysbiosis and a niche permissive to periodontitis.

**Conclusion:**

The diet–oral microbiome–systemic inflammation axis is bidirectional and clinically relevant. Understanding both direct ecological regulation and indirect metabolic effects is essential to support precision nutrition strategies aimed at maintaining oral microbial balance and systemic inflammatory risk mitigation.

## 1 Introduction

The oral microbiome is acquired as early as intrauterine life. Several studies have demonstrated the presence of microorganisms in the placenta, umbilical cord blood, amniotic fluid, and meconium. Notably, bacteria found in the placenta, umbilical cord blood, amniotic fluid, and meconium of the fetus resemble those found in the oral cavity rather than those in the vagina or gastrointestinal tract of the mother (1–5). At birth, a substantial portion of the newborn’s oral microbiota is obtained through swallowing fluids from the birth canal and the maternal perianal region, or from the surrounding skin in the case of cesarean delivery (6). From this point onward, the oral and gastrointestinal microbiomes of the newborn remain similar until approximately 2 weeks of life, after which they begin to diverge (7).

Based on 16S rRNA gene comparative analyses, the oral cavity in adults has approximately 687 predominant species, distributed across niches, such as tongue, cheeks, tooth surfaces, gingival pocket, and palatine tonsils, depending on the availability of nutrients, oxygen levels, pH, and temperature, among other factors (8). Approximately 400–500 taxa have been detected in the periodontal pocket, a virtual space formed between the tooth and the free gingival margin (9, 10), with the remaining taxa distributed on the surfaces above, forming a highly organized, proliferative, and enzymatically active biofilm (9–13).

The microbial species comprising this microbiota exist in constant equilibrium with one other and with host’s defenses (symbiosis). Collectively, the bacteria respond to environmental stimuli having a certain capacity to “control” and can correct the disturbances of low or medium intensity (14). However, when these factors exceed the “resilience” capacity of the biofilm, the equilibrium is lost, causing dysbiosis.

Dysbiosis results in conditions that allow pathogenic bacteria, initially present in low numbers (pathobionts), to proliferate and increase virulence through mechanisms dependent on bacterial cell density and thus induce different pathologies, such as caries, periodontitis, peri-implant diseases, peri-implant mucositis, and candidiasis (15–18). Resolution of these pathologies that respond to the dysbiosis model not only considers therapeutic actions focused on limiting and repairing the damage but also entails the need for restoration of the microbial balance and reestablishment of a health-compatible biofilm (19).

In addition to the host immune response, other extrinsic factors also modify the amount and composition of the oral biofilm, including tobacco use, poor oral hygiene, dentures, antibiotic exposure, human immunodeficiency virus infection, immunosuppression, diabetes, and radiotherapy (19–24). Although it is well established that diet affects oral health and that the consumption of refined sugars is associated with a high prevalence of dental caries, there is limited evidence regarding the impact of lifestyle-associated diets, such as caloric restriction, vegetarian, or vegan diets on oral health. This issue is particularly relevant given the accumulating evidence regarding the association between dietary habits and higher life expectancy, a lower incidence of chronic non-communicable diseases, a lower risk of cancer, activation of molecular pathways associated with longevity, and a reduction of co-morbidities mediated by chronic systemic inflammation, which result from a typical Western omnivore diet (25–30).

Dietary effects on the oral microbiome are evident throughout the entire lifespan. It has been observed that breastfed infants already display the acquisition of *Lactobacillus* sp., known for their antimicrobial properties, as early as 3 months of age, whereas formula-fed infants acquire them later (31, 32). Thus, diet plays a critical role in the composition of the oral microbiome, which is an important aspect to be considered, given the relevance of the oral microbiome in the first months of life, when it participates in the stimulation and tolerance of the oral mucosa immune system (33), as well as in adulthood when the characteristics of the oral biofilm are closely related to systemic health due to its role in inhibiting potential pathogens, in regulating immune responses, in nutrient absorption, and also in metabolism (34).

## 2 Methodology

This narrative, non-systematic review was designed to map mechanistic insights and summarize emerging evidence on the relationship between diet, the oral microbiome, and systemic inflammatory and metabolic health. A broad literature search was conducted, focusing on peer-reviewed studies that reported dietary exposures (e.g., caloric restriction, plant-based dietary patterns, nitrate and fiber intake, and high-sugar or high-fat processed diets) and their effects on oral microbial ecology and systemic inflammation-related outcomes. Searches were conducted in major biomedical databases using thematic keyword combinations related to diet, oral microbial niches, dysbiosis, oxidative stress, systemic inflammatory biomarkers, and periodontitis-associated comorbidities. The inclusion criteria prioritized original research and clinical trials describing biological mechanisms, ecological shifts, host-microbiome signaling, metabolite outcomes, or systemic clinical proxies. No restrictions were applied to study design or geographic region when studies were informative for mechanistic context.

Articles were screened for scientific relevance by evaluating study aims, microbiome-related endpoints, biological plausibility, and contributions to understanding the diet–microbiome–systemic inflammation axis. Evidence was qualitatively synthesized and categorized by predominant diet type and biological impact, with emphasis on interconnections between oral ecological stability, inflammatory modulation, metabolic disruption, and systemic disease risk markers. Given the exploratory nature of the review, study heterogeneity was anticipated and considered as a source of expanded insight rather than a basis for exclusion. Findings were integrated to provide a mechanistic framework and to identify knowledge gaps regarding how diet influences oral microbiome homeostasis directly and indirectly through the systemic inflammatory and metabolic pathways.

## 3 Effect of caloric restriction on the oral microbiome

Caloric restriction is defined as a reduction in energy intake without malnutrition, well below the number of calories that would be consumed *ad libitum*. This dietary feeding mode is associated with delayed cardiovascular aging and helps to prevent atherosclerotic cardiovascular disease (CVD) (35).

There is scarce evidence regarding the effect of caloric restriction on the oral microbiome. Raynold et al. have studied a cohort of 81 rhesus monkeys (*Macaca mulatta*) in which the effects of long-term caloric restriction on the extent and severity of naturally occurring chronic periodontal disease, local inflammatory and immune responses, and periodontal microbiology were assessed. It was found that, in male monkeys subjected to dietary caloric restriction, the local inflammatory response against chronic bacterial biofilms was reduced, showing lower levels of inflammatory markers (IL-1β and IL-8), lower levels of markers of periodontal destruction (probing depth and clinical attachment level), lower levels of local oxidative stress and *β*-glucuronidase in crevicular gingival fluid, and lower levels of IgG, which indicate that caloric restriction could control the antibody response to periodontal pathogenic biofilms (36).

A recent non-randomized clinical trial (*n* = 14) studied the effects of caloric restriction through intermittent fasting on the subgingival microbiota in overweight and obese individuals. Although the study did not observe significant changes in the microbial profile after 6 months of intervention, participants exhibited a reduction in gingival blood pressure and a decrease in probing pocket depths (PPD) of 4–5 mm, which suggested that, despite the limited number of participants, caloric restriction may exert an anti-inflammatory effect.

## 4 Vegan/vegetarian diet and oral microbiome

Evidence support the impact of protein consumption on the prevalence of cardiovascular disease, type 2 diabetes, and cancer. Studies published primarily in North America and Switzerland have demonstrated a strong positive relationship between higher animal protein intake and a higher mortality due to chronic non-communicable diseases. In contrast consumption of plant-derived proteins reduces the risk of chronic pathologies, and in groups with reduced protein intake, there was a favorable impact on longevity and age-related diseases (25–30).

Regarding plant-based diets and diets low in animal protein, one of the most studied parameters is the effect of inorganic nitrate intake on the oral microbiota. This compound has emerged over the last decade as potentially beneficial to cardiovascular health (37, 38), with green leafy vegetables such as rocket (arugula), spinach, kale, certain types of lettuce, and beetroot being the main sources of inorganic nitrate (38).

Of the ingested inorganic nitrate that is absorbed and enters the circulation, 25% is excreted into the oral cavity by the salivary glands, where facultative anaerobic bacteria reduce salivary nitrate to nitrite by the action of nitrate reductases (39). This nitrite is then reduced to nitric oxide in tissues and organs, enhancing the bioavailability of this important vasodilator compound that is associated with the regulation of endothelial function and decreased blood pressure (40). Vegetarian diets are associated with lower blood pressure, which has been suggested to be related to greater nitrate consumption. The depletion of oral bacteria by the use of antibacterial mouthwash has been shown to disrupt the oral nitrate–nitrite pathway and to reduce plasma nitrite levels, which is associated with an increase in blood pressure in healthy individuals and those with hypertension (41–44).

Evidence shows that, in young, healthy vegetarians, and omnivores with similar characteristics, the use of antibacterial rinses reduces the activity and abundance of nitrate-reducing species in the oral cavity, particularly in omnivores, thereby supporting the hypothesis that vegetarian diets could be an interesting tool to amend changes in the oral microbiome associated with antibacterial and/or antibiotic mouthwash treatments, which can trigger other comorbidities (45).

A study that evaluated the effects of direct nitrate supplementation in young people between 18 and 22 years and in older adults 70 to 79 years reported a variation in the salivary microbiome compared with placebo on nitrate supplementation. The results showed an increase in the relative abundance of bacteria with a greater ability to reduce nitrates, with higher abundances of *Rothia* and *Neisseria* and lower abundances of *Prevotella* and *Veillonella*, in addition to reporting higher plasma levels of nitrate and nitrite associated with lower blood pressure in the same study groups (46).

The vegan diet analysis shows that the human oral microbiota of vegans differs significantly from that of omnivores, both in terms of community structure and taxonomic composition, as well as in the genomic potential of the bacterial community (30). Some studies have reported smaller differences between urban-dwelling vegans and omnivores compared with the more significant differences described in other publications comparing African agriculturalists and hunter-gatherers, characteristics that would reflect differences in diets. This finding may be related to lifestyles within an urban lifestyle factors such as limited access to pesticide-free vegetables, water quality, xenobiotic agents, and other factors that could be modulating the oral microbiome (47, 48).

Similarly to the gastrointestinal microbiome, in which early-life exposure is a prerequisite for the diet to exert a profound effect on the microbial community— for the adaptation of the microbiota to dietary patterns with a loss of recovery potential over generations (49)—the oral microbiota could also be subjected to such constraints. This may explain why no differences in microbial diversity between vegans and omnivores were observed despite a median duration of 5.5 years (range 1–26 years) of adherence to a vegan diet. Notably, all vegan participants had changed dietary habits during or after their second decade of life (age of vegan debut 10–57 years) (47).

Another important aspect is that certain dietary components could influence the oral microbial community. Fiber intake, medium-chain fatty acids (MCFAs), such as caprylic, capric, and lauric acids, monounsaturated fatty acids (MUFAs), such as nervonic and cetoleic acids, and poly-unsaturated fatty acids (PUFAs), such as docosahexaenoic acid, docosapentaenoic acid, eicosapentaenoic acid, and stearidonic acid, are associated with bacterial diversity, community structure, and abundance in the oral cavity. The antimicrobial properties of fatty acids have been well recognized, with MCFAs, omega-3 PUFAs, and omega-9 MUFAs shown to have antimicrobial effects against oral microbiota *in vitro* (50–55).

## 5 Sugar intake and effect on the oral microbiome

Regarding carbohydrate consumption, it has been reported that high glucose levels in the saliva of subjects with diabetes and pre-diabetes could disturb the oral environment, enhancing the growth of some bacterial species at the expense of others (56, 57). In addition, the concomitant reduction in saliva secretion in diabetes may result in a reduction of microbial diversity (58), and hyperglycemia may lead to the acidification of the oral cavity, disturbing the homeostasis of the oral microbiota (59). Saeb et al. evaluated the proportion of non-pathogenic, pathogenic, and beneficial/probiotic bacteria and found that, in normoglycemic individuals, 73.3% of the operational taxonomic units analyzed (OTU) were not oral pathogens, 17.4% were oral/dental pathogens, and 9.3% were beneficial probiotic bacteria; in the case of impaired glucose tolerance, only 46.7% were not oral pathogens, 33.3% oral/dental pathogens, and 20% were beneficial or probiotic bacteria. In contrast, in those with diabetes, 61.54% were not oral pathogens, 38.46% were oral/dental pathogens, and no beneficial probiotic bacteria were identified. The absence of beneficial bacteria could be associated with an increase in pathogenic microorganisms, which, in turn, could be associated with the presence of molecules that could inhibit the growth of probiotic bacteria (60–62).

Similar to what happens in patients with diabetes, where certain diets favor the development of a systemic condition that controls the oral microbiome, in obesity and metabolic syndrome, the diversity and structure of the oral microbiome are also significantly different from that of normal-weight individuals. Changes in the function and structure of the oral microbiome in obese individuals may indicate greater susceptibility to oral pathologies, with higher levels of markers of systemic inflammation and, at a local level, higher levels of markers associated with periodontal disease (61, 62). In addition, it has been observed that the periodontal microbiome of obese individuals displays a significantly lower diversity and bacterial richness, compared with normal weight individuals, with a greater abundance of the genera *Prevotella*, *Granulicatella*, *Peptostreptococcus*, *Solobacterium*, *Catonella*, and *Mogibacterium*, and less abundance of the genera *Haemophilus*, *Corynebacterium*, *Capnocytophaga*, and *Staphylococcus* (63).

Based on the aforementioned and considering the limited evidence, new perspectives have emerged on the association between diet and oral bacteria, including the hypothesis that the diversity of microbiota present in the cavity could be modulating the perception of taste, constituting a possible factor associated with children developing obesity (64).

## 6 Micronutrients

Diet not only influences the oral microbiome, but the oral microorganisms also influence the efficiency with which the nutrients are metabolized. Pathways involved in the metabolism of some amino acids that are less abundant in vegan diets were enriched in vegans, potentially reflecting a competitive advantage for bacteria better equipped at utilizing amino acids in an environment where the substrate is scarce (47).

Additionally, individuals following a vegan diet had an enriched bacterial genomic potential for production of biotin (vitamin H or vitamin B7), which is involved in gene regulation and human health, growth, and development (65). Furthermore, biosynthesis of pantothenate (vitamin B5 or the “anti-stress vitamin”) is a precursor of coenzyme A, an essential molecule in microorganisms, required for blood cells synthesis and wound healing. The deficiency of vitamin B5 results in reduced cortisol production, increased arthritic pain, myalgia, fatigue, headache, depression, insomnia, and widespread “proinflammatory” effects on the immune system (66). In vegans, the potential for folate biosynthesis was reduced, mirroring lower levels of biotin and pantothenic acid and higher levels of dietary folate.

Moreover, available evidence suggests that micronutrients such as vitamins play an important role in preventing and treating oral diseases, including periodontitis (67). Similarly, some fatty acids, such as omega-3, participate as a substrate for the synthesis of resolvins and protectins—biomolecules associated with resolving inflammation and repairing tissue in the oral cavity (68–70).

## 7 Conclusion

**Figure 1:**
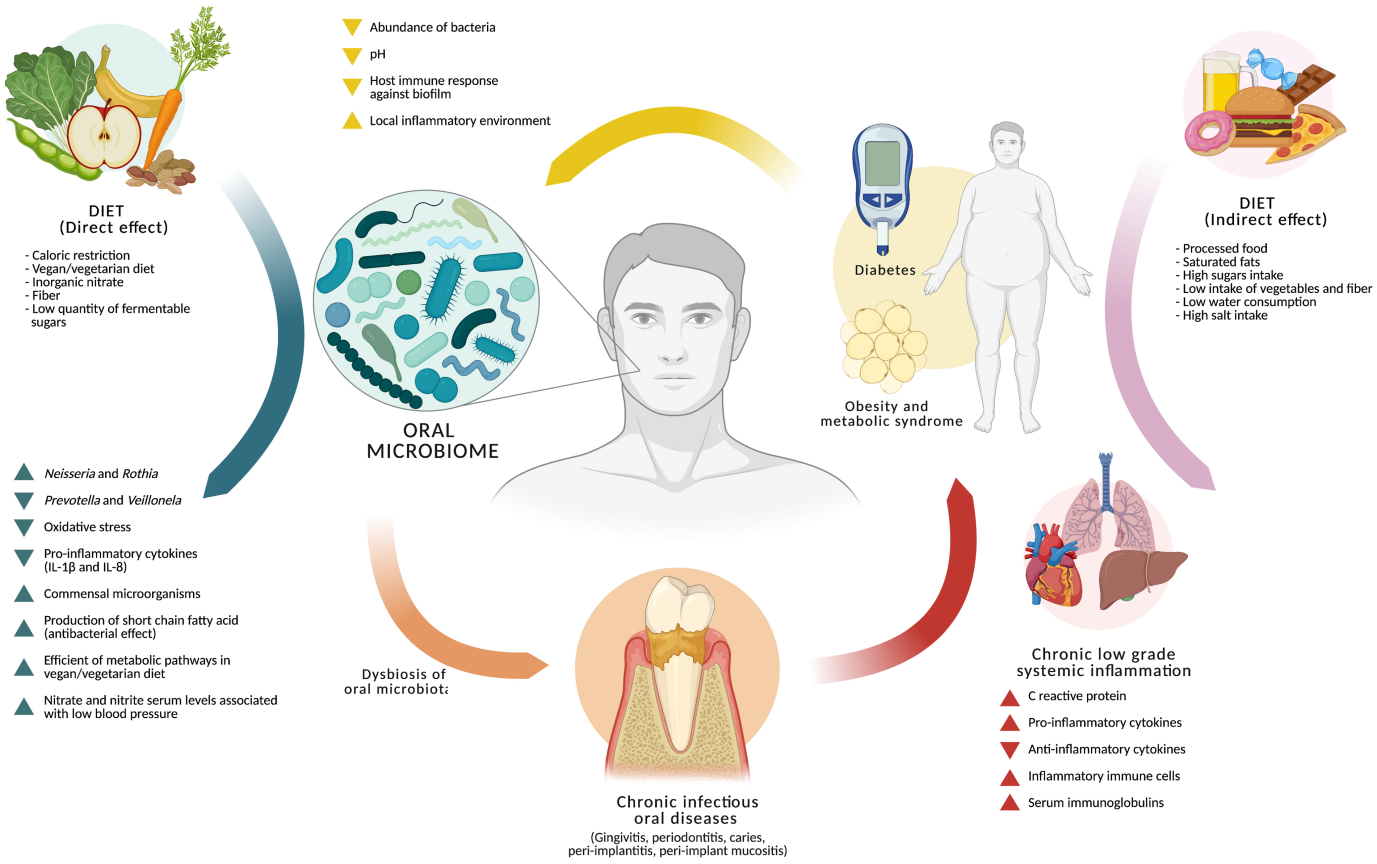
Model of the effect of diet on the oral microbiome. The diet from caloric restriction, vegan/vegetarian diet, inorganic nitrate intake, fiber consumption, and low quantity of fermentable sugars, would have a protective effect on the oral microbiome by modulating, in favor of the host, the number of commensal microorganisms, the abundance of *Prevotella* and *Veillonela*, and the production of short-chain fatty acid. In addition to reducing the levels of inflammation and oxidative stress, it could influence systemic parameters such as nitrate and nitrite serum levels associated with low blood pressure. However, there is an “indirect” or systematic pathway through which diet would influence the oral microbiome, which is related to a diet high in processed foods, a high consumption of saturated fats and sugars, and a low intake of vegetables and fibers, which favors the development of systemic pathologies, mainly chronic non-communicable diseases that influence conditions in the oral cavity (low pH, high sugar levels, lower host immune response, etc.,) favoring microbial growth at the expense of others. This promotes dysbiosis of the oral microbiome and the development of oral pathologies such as caries, or other disorders associated with the supporting and protective tissues of the tooth (gingivitis, periodontitis, peri-implantitis, and peri-implant mucositis), some of which are strongly linked to systemic inflammatory potential, which could exacerbate and/or perpetuate existing conditions largely determined by the host’s lifestyles.

Current evidence suggests that diet influences the oral microbiome through intertwined direct ecological pressures and indirect metabolic and inflammatory remodeling, although substantial uncertainties remain. Evidence derived from human and animal models shows that plant-rich diets, high fiber intake, and inorganic nitrate exposures may support a more resilient microbial ecosystem by supporting commensal taxa, reducing oxidative stress, and dampening local pro-inflammatory cytokine expression. However, these studies remain highly heterogeneous in design, population characteristics, follow-up time, and microbial endpoints, limiting the ability to infer consistent long-term ecological adaptation or causality. Data from caloric restriction models suggest reduced local inflammatory destruction and possible modulation of antibody responses against dysbiotic biofilms, but sample sizes in clinical studies are markedly small, and findings across trials are inconsistent. Furthermore, several human vegan cohorts show no clear diversity advantage when dietary debut occurs after the second decade of life, raising questions about microbial memory, niche entrenchment, and generational irreversibility in the oral cavity, analogous to constraints described in the gut microbiome. Collectively, these findings imply that diet may not independently increase microbial richness but instead act to protect against disease-driven collapse of microbial balance, sustaining resilience rather than expand diversity (Figure 1).

In contrast, pro-inflammatory processed diets are reliably linked to the development of systemic metabolic conditions, including impaired glucose tolerance, obesity, and metabolic syndrome, which remodel salivary proxies (pH, glucose concentration, and flow), fostering a dysbiotic habitat permissive to inflammatory oral disease progression. In established periodontitis, particularly in severe stages, systemic inflammatory amplification becomes evident, but temporal precedence remains unclear: whether dysbiosis predates systemic inflammation or is fueled by systemic metabolic breakdown requires targeted longitudinal evidence.

Available evidence supports a biologically plausible, predominantly indirect diet–microbiome pathway, in which systemic metabolic and inflammatory states act as major effectors of oral dysbiosis and periodontal destruction. Direct dietary modulation of the oral microbiome shows promising protective signals but lacks conclusive long-term and human interventional validation. Thus, diet may function less as a driver of *de novo* microbial expansion and more as a strategy to preserve ecological stability, enhance resilience, and mitigate inflammatory burden, locally and systemically.

To advance the field, new research should prioritize (1) longitudinal and life-course cohorts capturing early diet debut to test oral microbial memory effects; (2) mechanistic multi-omics studies linking nitrate, fiber, and fatty acid metabolite outputs in saliva, gingival crevicular fluid, and serum inflammatory profiles; (3) controlled dietary interventions targeting salivary glucose reduction, pH buffering, and microbial resilience rather than pathogen depletion alone; (4) host-microbe signaling metrics describing cytokine tone, oxidative stress, microbial network topology, and antibody shaping; and (5) systemic clinical proxies such as endothelial, glycemic, and inflammatory markers integrated with oral microbial function. Moreover, exploration of the oral microbiome–taste perception interface may unlock novel preventive hypotheses in childhood obesity. Integrating direct ecological regulation with systemic inflammatory mediation will be essential to inform precision nutrition strategies targeting oral microbial homeostasis and systemic inflammatory risk mitigation.
